# Case of Hydrothorax

**Published:** 1803-05-01

**Authors:** 

**Affiliations:** Member of the Royal College of Surgeons, London


### Case of Hydrothorax, by Mr. Low, *Member of the Royal College of Surgeons, London.*

MRS. R. No. 47, Bell Street, Paddington, aged fifty-three, of a robust stature, with a short neck, and of a sanguineo phlegmatic temperament, had throughout her life enjoyed a very good state of health, (except having experienced, at different periods, several rheumatic and slight arthritic attacks) until a short time previous to the 18th of August last, at which time I first visited her; she then complained of violent pains of the upper and lower extremities, the joints of which were much tumefied, the legs even anasarcous; the pulse was frequent and irregularly intermittent; the bowels were costive, the tongue was white, with thirst and other febrile symptoms. I ordered an electuary with c. tartar. Pulv. jalap. & elect c. senna to be taken occasionally, to keep the bowels open; a saline diaphoretic mixture to be taken every sixth hour, with a pill containing calom. ppt. gr. j. pulv. ant. Lond. gr. iij. and a stimulating linniment to be rubbed upon the pained tumid joints twice a day. By persevering in this plan, in less than three weeks, the fever, and the pain and swelling of the joints, which I then supposed to be the most urgent symptoms, abated. My patient at this period informed me, that she had, for six months past, felt great inconvenience in lying upon her left side, and which she now found quite impracticable; her respiration had also become very laborious; she started frequently, and awoke with considerable disturbance from her sleep, and was obliged to lie with her head very much elevated; the pulse was still frequent and irregularly intermittent; the legs also continued to be much swoln, receiving the impression of the fingers when applied to them; the urine was high coloured and sparing in quantity, and the bowels were costive, except when the electuary was taken. No undulation could be perceived upon shaking the thorax or striking it with the fingers, which sensation, although it has been laid down by almost every author who has written upon upon the subject, as regular in its occurrence, I have never yet been able to detect except in empyema, where one side of the lungs has been almost totally absorbed. In this state of the disease, after applying a large blister upon the right side of the thorax, I directed twenty-five drops of the saturated tincture of digitalis (obtained from Apothecary's Hall) to be taken three times a day, and in the space of a fortnight, a very considerable abatement was perceptible in every symptom. The pulse had become much more regular, and was reduced in frequency from 110 to 64 pulsations in a minute; the oppression of the chest and difficult respiration were considerably relieved, the swelling of the legs had also very much subsided; she now could enjoy several hours sleep, which she had not done for six months previously, and was capable of taking gentle exercise about the room. I now ordered the dose of tincture of digitalis to be increased to thirty drops three times a day, which was persisted in for about three weeks longer, by which time my patient perfectly recovered; no interruption could now be perceived in the pulse; she could sleep well through the night, could lie down on either side without suffering any inconvenience, and was capable of attending to every domestic occupation.

I have exhibited the digitalis in several cases similar to the above, most of which have been attended with nearly an equal degree of success. I have also given the digitalis in several cases of ascites, but not with that success which almost invariably has occurred from its exhibition in hydrothorax.

Does the efficacy of digitalis depend upon its power of retarding the accelerated action of the arterial system, thereby causing its secretions to be diminished, and thus giving an opportunity to the absorbents to take up the accumulated fluid, which may be deposited by a retrograde action (as explained by Dr. Darwin in his Zoonomia) either into the stomach, intestines, kidneys, or bladder, causing vomiting, catharsis, or diabetes?

*Tavistock Row, Covent Garden.*

*April* 2. 1803.

REPORT

